# Clinical predictors of shunt response in the diagnosis and treatment of idiopathic normal pressure hydrocephalus: a systematic review and meta-analysis

**DOI:** 10.1007/s00701-021-04922-z

**Published:** 2021-07-08

**Authors:** Santhosh G. Thavarajasingam, Mahmoud El-Khatib, Mark Rea, Salvatore Russo, Johannes Lemcke, Lana Al-Nusair, Peter Vajkoczy

**Affiliations:** 1 Faculty of Medicine, Imperial College London, London, United Kingdom; 2 Department of Neurosurgery, Imperial College Healthcare NHS Trust, London, United Kingdom; 3 Department of Neurosurgery, Unfallkrankenhaus Berlin, Berlin, Germany; 4 Department of Neurosurgery, Charité-Universitätsmedizin Berlin, Berlin, Germany

**Keywords:** Normal pressure hydrocephalus, Diagnosis, Shunt response, Shunt, Predictors

## Abstract

**Background:**

Positive shunt response (SR) remains the gold standard for diagnosing idiopathic normal pressure hydrocephalus (iNPH). However, multiple pathologies mimic iNPH symptoms, making it difficult to select patients who will respond to shunt surgery. Although presenting features, extended lumbar drainage (ELD), infusion test (IT), intracranial pressure monitoring (ICPM), and tap test (TT) have been used to predict SR, uncertainty remains over which diagnostic test to choose.

**Objective:**

To conduct a systematic review and meta-analysis to identify clinical predictors of shunt responsiveness, evaluate their diagnostic effectiveness, and recommend the most effective diagnostic tests.

**Methods:**

Embase, MEDLINE, Scopus, PubMed, Google Scholar, and JSTOR were searched for original studies investigating clinical predictors of SR in iNPH patients. Included studies were assessed using the QUADAS-2 tool, and eligible studies were evaluated using univariate and bivariate meta-analyses.

**Results:**

Thirty-five studies were included. Nine studies discussed the diagnostic use of presenting clinical features, 8 studies ELD, 8 studies IT, 11 studies ICPM, and 6 studies TT. A meta-analysis of 21 eligible studies was conducted for TT, ELD, IT, and ICPM. ICPM yielded the highest diagnostic effectiveness, with diagnostic odds ratio (DOR) = 50.9 and area under curve (AUC) = 0.836. ELD yielded DOR = 27.70 and AUC = 0.753, IT had DOR = 5.70 and AUC = 0.729, and TT scored DOR = 3.86 and AUC = 0.711.

**Conclusion:**

Intraparenchymal ICPM is statistically the most effective diagnostic test, followed by ELD, IT, and lastly TT. Due to the higher accessibility of TT and IT, they are recommended to be used first line, using a timed-up-and-go improvement ≥ 5.6 s or a Rout cut-off range between 13 and 16 mmHg, respectively. Patients who test negative should ideally be followed up with ICPM, using mean ICP wave amplitude $\ge$ 4 mmHg, or 1- to 4-day ELD with an MMSE cut-off improvement $\ge$ 3. Future research must use standardized methodologies for each diagnostic test and uniform criteria for SR to allow better comparison.

## Introduction

Normal pressure hydrocephalus (NPH), a syndrome discovered by Hakim and Adams [1], classically presents with dementia, gait disturbance, and urinary incontinence [23]. Idiopathic NPH (iNPH) is the most common form of adult-onset hydrocephalus, and the current gold standard for diagnosing iNPH is shunt response (SR), which is also the treatment [21, 23, 37, 45, 51, 65]. Although clinical improvement has been reported in up to 90% of patients following shunt surgery [34], this value has also been as low as 46.7% [5]. For example, Hebb and Cusimano [23] found that 59% of iNPH patients improved post-shunt, with long-term improvement reported in only 29%. This disparity in reported outcomes reflects the difficulty in selecting suitable patients for shunt surgery as many pathologies mimic iNPH symptoms [41, 43, 57].

Existing iNPH guidelines, such as the widely cited Japanese iNPH guidelines (2012) [44], a narrative review, outline various clinical tests that can be used in aiding iNPH diagnosis. Alongside Hakim’s triad, radiological and biochemical markers, these include the tap test (TT), infusion test (IT), extended lumbar drainage (ELD), and intracranial pressure monitoring (ICPM) [44]. However, the current guidelines [44] do not include explicit diagnostic parameters and cut-off values for each test. Hence, in practice, there is a lack of consistency in the method and evaluation of each test.

The current literature is outdated with regard to the analysis of the diagnostic tests and the presenting factors which may predict SR. In the most recent literature review, Nunn et al. [48] was published last year and investigated the accuracy of ELD. This review [48] included 4 papers of which only one was published after 2003. Furthermore, the last systematic reviews to investigate the diagnosis of iNPH were Relkin et al. (2005) [56] and Hebb and Cusimano (2001) [23]. While both investigated multiple iNPH diagnostic features, there was little comparison within and between diagnostic tests, and no meta-analysis was performed [23, 55]. Therefore, to fill this gap in the literature, this review aims to evaluate the diagnostic effectiveness of presenting features, TT, IT, ELD, and ICPM, by incorporating the latest primary research.

## Methods

### Literature search

This systematic review was conducted following the Cochrane Collaboration guidelines [9] and Preferred Reporting Items for Systematic Reviews and Meta-Analyses (PRISMA) [42]. A comprehensive search of MEDLINE, Embase, and Scopus was conducted from January 2003 to November 2020. January 2003 was chosen to encompass the literature after the end search year of Relkin et al. [56], the last seminal review on iNPH diagnosis. The search string consisted of the search term “Normal Pressure Hydrocephalus”. Additional articles were identified through manual searches on PubMed, Google Scholar, and JSTOR.

### Study inclusion and exclusion criteria

Original articles in the English language that reported SR in relation to diagnostic tests were included. Our study selection criteria included the following: adult iNPH patients, radiological confirmation of hydrocephalus, 1 or more clinical features of NPH, use of cerebrospinal fluid (CSF) shunt, objective system of functional grading of patients preoperatively and a minimum of 3 months postoperatively, and that the diagnostic test was evaluated for the ability to predict SR. Radiological and biochemical studies were excluded, as the depth of literature on the role of both of these tests in iNPH diagnosis warrant separate meta-analyses.

### Eligibility assessment, data extraction, and quality assessment

Studies were evaluated for eligibility independently by two reviewers. Disagreements were resolved by consensus after discussion with a third reviewer. Data were extracted using the Covidence data collection tool [10]. All papers were critically appraised using the Quality Assessment of Diagnostic Accuracy Studies (QUADAS)-2 tool [69].

### Statistical analysis

An Egger’s regression and asymmetry test [9] was used to assess publication bias (p < 0.05% = significant). A univariate analysis yielded the log diagnostic odds ratio (logDOR) for each diagnostic test and the DerSimonian-Laird (DSL) summary point, visualized in forest plots. The diagnostic odds ratio (DOR) was calculated by taking the natural logarithm of the logDOR. Cochrane’s Q test [9] (p < 0.05% = significant) and Higgins I^2^ test [68] (heterogeneity: < 25% = low; 25–50% = moderate; > 50% = high) were used to assess heterogeneity. Finally, a bivariate analysis, which is known to give a slightly more accurate estimation of diagnostic performance than univariate analysis in small sample size analyses, was utilized to plot a summary receiver operating characteristic (SROC) curve. An area under curve (AUC) of 1 indicates perfect diagnostic effectiveness; an AUC $\le$ 0.5 indicates an ineffective test. Statistical significance was assumed for *p* < 0.05. Statistical analysis was carried out by utilizing the mada [11] and meta [60] packages with the R software (version 4.0.4) [55].

## Results

**Fig. 1:**
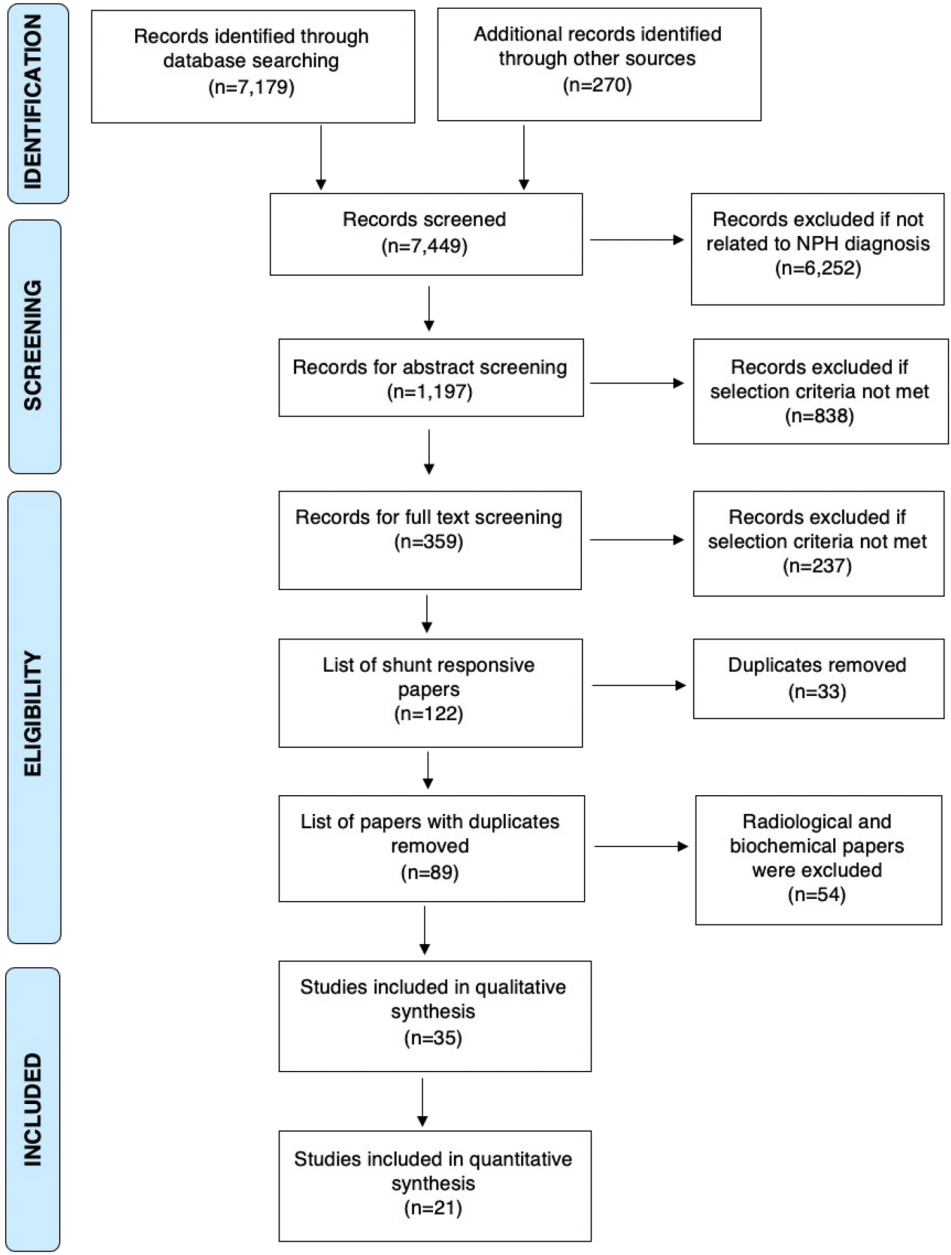
Preferred Reporting Items for Systematic Reviews and Meta-Analyses (PRISMA) [42] flowchart outlining the study selection process for qualitative synthesis (systematic review) and quantitative synthesis (meta-analysis)

**Fig. 2:**
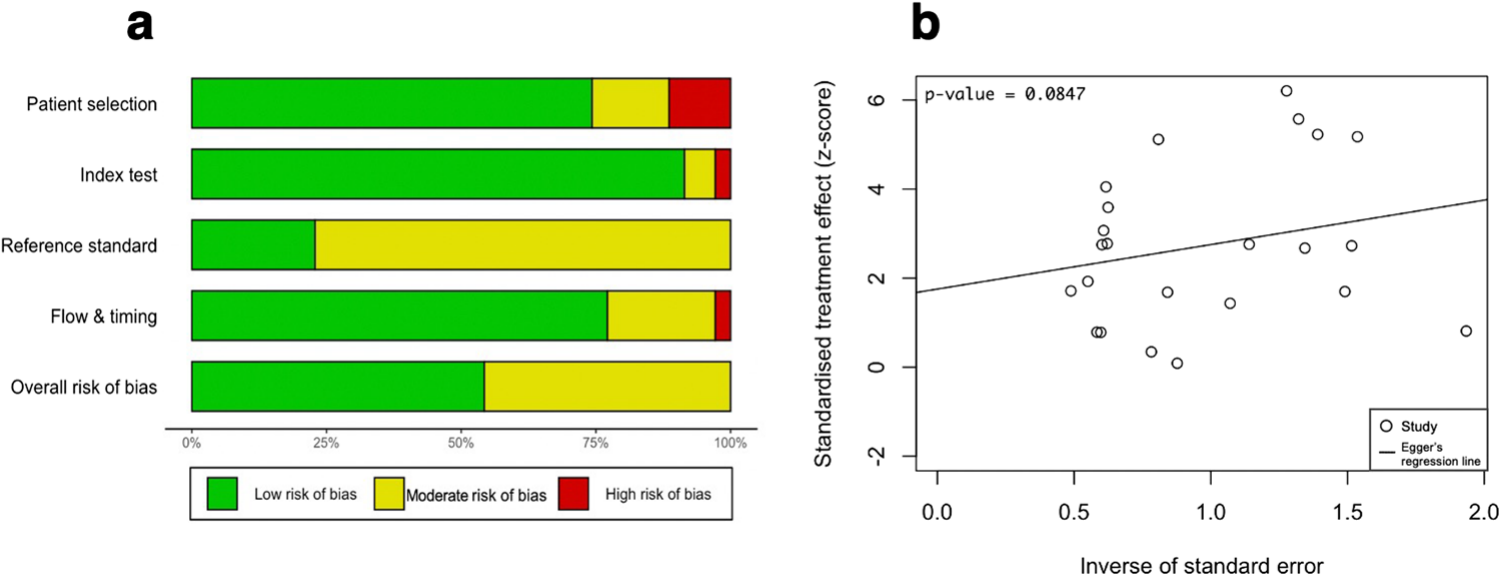
In Fig. 2a, risk of bias summary plot with bar chart of the distribution of risk-of-bias judgments for all included studies (n = 35) across the domains of the QUADAS-2 tool [69], shown in percentages (%), is shown. In the bottom, an overall risk of bias, which represents the collated risk-of-bias judgments for all domains, is depicted. The reference standard refers to shunt responsiveness. In Fig. 2b, an Egger’s asymmetry test funnel plot [9] of all data points included in the meta-analysis (n = 24; two studies used twice due to discussing two index tests) indicating presence and degree of publication bias is shown. P-value < 0.05 is deemed significant and implicates publication bias. Egger’s asymmetry test yielded 0.0847%, calculated running an Egger’s regression [9] (see Egger’s regression line) on the collated logDOR and standard errors of all data used in the meta-analysis (n = 24)

The literature search retrieved a total of 7179 papers, of which 359 papers underwent full-text review and 35 studies were included (Fig. 1). The QUADAS-2 tool [69] scored all included studies at low to moderate risk of bias overall, none scoring high risk (Fig. 2a). No significant publication bias was detected by Egger’s test (p = 0.0847) (Fig. 2b) [9]*.*

### Presenting clinical features

Nine studies investigated the effect of presenting symptomology and patient characteristics on SR (Table 1) [3, 30, 35, 36, 38, 39, 44, 50, 61].

**Table 1:** Presenting clinical features

Study	Shunted patients	Methodology	Criteria for SR	Main reported outcomes
Thomas et al. (2005) [63]	n = 4 2	• Assessments: MMSE, WMS, ROCF, RAVLT, line-tracing trail-making test B, Stroop color ward • Follow up: 3 months	• 4-point improvement in MMSE • Or improvement by 1 SD in 50% of all neurocognitive subtests	• WMS Immediate recall: OR: 0.250 (CI: 0.06–0.95), p = 0.042. Patients with 1SD below population average are 4.0 × less likely to respond • WMS Immediate recall AND ROCF direct copy: OR: 0.165 (CI: 0.02–0.94) p = 0.042. Patients with 1SD below population average in both tests are 6.1 × less likely to respond • WMS immediate recall AND Stroop color word: OR: 0.151 (CI: 0.02–1.03) p = 0.054. Patients with 1SD below population average in both tests are 6.6 × less likely to respond
Mahr et al. (2016) [37]	n = 31	• Assessments: Kiefer score, SLHS, MMSE, standardized gait testing, grooved pegboard test, and mRS • Follow-up: 12 months	• Both excellent and improved were classified as responders: • Excellent (relief of all symptoms) • Improved (Kiefer score reduction of at least 10%)	• mRS and SLHS scores were on average higher in non-responders • Kiefer scores were higher in non-responders than responders. Cut-off of > 5 points for positive SR: sensitivity = 58%, specificity = 71%, PPV = 88% and NPV = 31%. Cut-off of > 9 points: sensitivity = 4%, specificity = 43%, PPV = 20%, and NPV = 11% • MMSE scores were higher in responders than non-responders (p = 0.043). A cut-off value of 21 MMSE points or greater for positive SR: sensitivity = 67%, specificity = 93%, PPV = 93%, and NPV = 67% • Mean age, CMI, and mean symptom duration of non-responders were 73.9 years and 2 and 9.7 months, respectively. For those improved: 67.7 years and 3 and 32.1 months, respectively. For excellent responders: 68.8 years and 2 and 18.5 months, respectively
Meier and Miethke. (2003) [41]	n = 200	• Assessments: new model consisting of both the Black grading scale for shunt assessment and the Kiefer and Steudel’s clinical grading scale • Follow-up: 7 months	• Excellent, improved, and fair were classified as responders using their new model	• Age, etiology, and symptomology have significant predictive values of shunt responsiveness • Early NPH (no cerebral hypertrophy) patients with symptom onset of less than 12 months before surgery had a more positive response than those with greater than 12 months (p = 0.01) • Presence of (p = 0.01) and severe (p = 0.01) dementia were indicators of poor prognosis. Patients with no memory symptoms fared better than patients with short-term memory problems who in turn had a better prognosis than those with acute dementia
Kazui et al. (2013) [30]	n = 100	• Assessments: mRS, MMSE, 3 m TUG, and NPH grading scale • Follow-up: 3, 6, and 12 months	• 1 or greater point improvement in mRS	• Factors likely to predict disappearance of gait symptoms: young age (OR: 0.88 [CI: 0.79–0.99] p = 0.032), low INPHGS gait score (OR: 0.36 [CI: 0.17–0.77] p = 0.008), low TUG (OR: 0.90 [CI: 0.84–0.96] p = 0.002) • Factors likely to predict disappearance of cognitive symptoms: no hypertension (OR: 0.50 [CI:0.19–1.30] p = 0.16), low iNPHGS cognitive score (OR: 0.47 [CI: 0.27–0.82] p = 0.007), high MMSE total score (OR: 1.10 [CI:1.02–1.20] p = 0.021), high memory subtest score (OR: 1.16 [CI: 1.01–1.34] p = 0.03), high visuoconstruction subtest score (OR: 8.44 [CI: 2.42–29.46] p = 0.001) Factors likely to predict disappearance of urinary symptoms: low iNPHGS urinary score (OR: 0.29 [CI: 0.15–0.57] p = 0.001)
Murakami et al. (2007) [46]	n = 24	• Assessments: Mori grading system, MMSE, and Barthel index • Follow-up: 10 to 36 months	• 1 rank improvement in ≥ 2 of the Mori grading triad components	• Young age was predictive for positive SR; mean age 75.8 for responder’s vs 79.9 for non-responders (p = 0.023) • 71.4% shunt responders and 20.0% non-shunt responders had no pre-existing causes, (p = 0.015). 80% non-responders had lacunas as opposed to 28.6% responders • No dominance in a particular triad domain was predictive of shut responsiveness
McGirt et al. (2005) [40]	n = 132	• Assessments: MMSE • Follow-up: 1, 3, 6 months and then yearly	• A 3-point or more improvement in the MMSE	• Predictive factors of SR: patients with gait disturbance as primary symptom (RR: 1.71 [CI: 0.42–6.91]) and shorter duration of iNPH symptoms (RR: 0.87 [CI:0.79–0.96]). Every additional year of symptom duration was associated with a 13% lower chance of treatment response Age, sex, vascular comorbidities, the presence or absence of any one of iNPH symptoms or the complete triad were not associated with SR
Poca et al. (2005) [52]	n = 56	• Assessments: NPH scale in the 3 triad domains. WMS, TMT part A and B, and MMSE • Patients were split into good and poor prognosis groups. The paper followed those in the poor group (idiopathic form, cortical atrophy, long disease evolution time, presence of dementia (MMSE < 24) and age > 64 years) • Follow-up: 6 months	• Moderate improvement: 1-point increase in NPH scale • Marked improvement: 2-point increase in NPH scale	• 21.4% of those shunted had at least 4 poor prognostic factors; 91.7% of these showed good response when shunted • All with gait dysfunction showed improvement and 90% with sphincter dysfunction showed improvement
Marmarou et al. (2005) [38]	n = 102	• Assessments: gait was assessed by giving patients several walking and sitting instructions. MMSE, Galveston orientation and amnesia test, controlled oral word association test, Benton visual retention test–revised, digit span forward and backward tasks, WMS and RAVLT • Follow-up: 12 months	• Patients and/or caregivers completed 10-day surveys looking at patient’s daily symptom status	• There was no significant relationship between SR and age. Of those > 75 years old, 94% responded, whereas of those < 75 years old, 94% responded • There was no significant difference between complete triad presentation and only 1 or 2 symptoms at presentation on shunt outcome. Of those > 75 years old: with full triad symptoms at presentation, 95% responded. Of those with 1 or 2 symptoms at representation, 95% responded. Of those < 75 years old: with full triad, 96% responded, with 1 or 2 symptoms, 90% 4responded
Bådagård et al. (2019) [3]	n = 332	• Assessments: modified Hellstrom iNPH scale, cognitive assessment section was excluded due to low patient participation • Follow-up: median 12.4 months	• An improvement of > 5 levels of mod-iNPH scale	• Age: those < 70 years old response rate was 62%, then 52%, and 39% for those 70–80 and > 80, respectively. Increasing age (CI: − 0.99 to − 0.28), p < 0.001) was a negative predictor (OR: 0.97 [CI: 0.93–1.01] p = 0.14) • Pre-op mod-INPH scale: a higher score was a predictor of better prognosis (CI: − 0.44 to − 0.19 p < 0.001) • Co-morbidities: ischemic stroke/TIA (CI: − 16.84 to − 3.29), p = 0.0038) were negative predictors • Longer waiting times before surgery were associated with less favorable outcomes (CI: − 1.44 to − 0.53 p < 0.001) • The following had no effect on SR: duration of symptoms (CI: − 0.10 to 0.059 p = 0.59); hypertension (CI: 7.17–2.93, p = 0.41); cardiovascular disease (CI: 8.35 to 4.8, p = 0.60); diabetes (B = 0.35 CI: − 5.17 to 5.88 p = 0.90); hyperlipidemia (CI: − 6.08 to 4.49, p = 0.77); thrombocyte inhibitors (CI: 0.67–2.14, p = 0.54); anticoagulants (CI: 0.30–3.13, p = 0.95)

Studies included assessing the use of clinical presenting features and patient characteristics in predicting shunt responsiveness. *SR*, shunt response; *MMSE*, mini mental state exam; *WMS*, Wechsler memory scale; *ROCF*, Rey-Osterrieth complex figure; *OR*, odds ratio; *NPH*, normal pressure hydrocephalus; *INPH*, idiopathic NPH; *SD*, standard deviation; *RAVLT*, Rey auditory visual learning test; *CMI*, comorbidity index; *INPHGS*, iNPH grading scale; *SLHS*, Stein and Langfitt hydrocephalus score; *mRS*, modified Rankin scale; *TUG*, timed up and go test; *TMT*, trail making test.

#### Symptom dominance

McGirt et al. [40] found that patients with gait disturbance as a primary symptom were twice as likely to respond than others, although complete triad presentation compared to the absence of one symptom did not affect SR. This was reinforced by Marmarou et al. [38] and Murakami et al. [46]. However, Meier and Miethke [41] found that the presence of dementia was significantly associated with a worse outcome (p = 0.01).

#### Symptom duration

Meier and Miethke [41] found that a symptom duration onset of under 12 months before shunt surgery was associated with a significantly better outcome. McGirt et al. [40] noted a 13% lower chance of positive shunt outcome for each additional symptom year before surgery. However, Bådagård et al. [3] and Mahr et al. [37] observed no effect of symptom duration on outcome.

#### Symptom severity

Bådagård et al. [3] found a higher modified iNPH scale score, which reflects less severe symptoms, to significantly predict better prognosis. Mahr et al. [37] saw an association between lower baseline Kiefer scores and SR; a cut-off of > 5 had a sensitivity = 58% and specificity = 71%. Meier and Miethke [41] found worsening and severe dementia to be indicators of poor SR. Thomas et al. [63] reported that those with one SD below the age-corrected population average for the following tests to fair 4–6 × worse when shunted: Wechsler memory scale (WMS) immediate recall and WMS immediate recall with Rey-Osterrieth complex figure direct copy or Stroop color word. Mahr et al. [37] found modified Rankin scale (mRS), Stein and Langfitt hydrocephalus score (SLHS), and mini mental state examination (MMSE) scores to be on average higher in responders than non-responders. Using an MMSE cut-off of ≥ 21 was reported to have a specificity = 93% and sensitivity = 67% [37]. Similarly, Kazui et al. [30] found those with a high MMSE total score to be 1.1 × more likely to respond. Overall, the worse the symptomology, the worse the prognosis.

#### Age

Mahr et al. [37] found the mean age in years of non-shunt responders to be 73.9 compared to 68.8 for excellent responders, while Murakami et al. [46] observed shunt responders mean age to be 75.8 and non-shunt responders 79.9. Bådagård et al. [3] split patients into 3 age groups; in those aged < 70, the response rate was 62% and then 52% and 39% for 70–80 and > 80, respectively. However, Marmarou et al. [38] and McGirt et al. [40] reported no significant relationship between age and SR.

#### Co-morbidities

Bådagård et al. [3] reported ischemic stroke and transient ischemic attack to be significant negative predictors of SR, while Kazui et al. [30] found patients with hypertension to be twice as likely not to respond to shunting. However, Bådagård et al. [3] reported that hypertension, cardiovascular disease, diabetes, hyperlipidemia, and anti-platelet and anticoagulant use had no significant impact on SR.

### Tap test

Six studies discussed the CSF TT as a predictor of SR using different criteria for TT response, outlined below (Table 2) [24, 26, 27, 64, 68, 71].

**Table 2:** Tap test

Study	Shunted patients	Methodology	Criteria for + ve TT	Criteria for SR	Side effects	Main reported outcomes
Shigeki Yamada et al. (2017) [73]	n = 151	• Assessments: TUG time in seconds was measured pre- and within 24-h post-TT. Percentage time improvement = (TUG time before TT minus TUG time after TT or shunt surgery)/TUG time before TT × 100 (%). Simple time difference = TUG time before TT – TUG time after TT or shunt surgery • TT involved ≥ 30 mL removal of CSF • Follow up: 3, 6, and 12 months	• TUG time improvement of ≥ 5 s	• TUG time improvement of ≥ 10 s for clinically sufficient out	• Not reported	• Post-TT: deteriorated (approx. 10% of patients), < 5-s improvement (≥ 50%), ≥ 5- to < 10-s improvement (24%), and ≥ 10-s improvement (13%) • Threshold of 5.6-s threshold predicts a shunt outcome improvement of ≥ 10 s in TUG 12-month post-shunt with sensitivity = 83.3%, specificity = 81.0% • TP = 21, FN = 4, FP = 4, TN = 40
Carsten Wikkelsø et al. (2012) [70]	n = 115	• Assessments: iNPH scale and mRS scale. iNPH Scale score: weighted mean score of assessments in gait, neuropsychology, balance, and continence. iNPH scale range: 0 to 100 (100 = normal performance in healthy people within an age range of 70–74 years) • TT involved 50 mL removal of CSF • Follow up: 12 months	• + 5% mean increase in motor and psychometric tests values compared to 24 h before	• + 5 points on iNPH scale • OR − 1 point on mRS	• Not reported	• 84% improved on iNPH scale. 69% improved on mRS scale (35% improved by 1 point, 25% by 2 points, and 7% by 3 points) • Significant correlation between gait tasks improvement (10 m of walking at free speed) with TT and improvement in iNPH score (r = 0.22, p = 0.02) • TT: sensitivity = 52%, specificity = 59%, PPV = 88%, NPV = 18% • TP = 51, FN = 47, FP = 7, TN = 10
Hertel et al. (2003) [24]	n = 15	• Assessments: pre- and post-TT patients were clinically assessed and evaluated using SPECT, pwMRI, neurological examination, walking test, psychometric examination (MMS), the HHS, and incontinence protocol • STT involved 40–50 mL removal of CSF • Follow up: 3 months	• Improved clinically, or by increasing perfusion in SPECT or pwMRI • − 4-point HHS scale • Imaging: global increase in cerebral blood flow on SPECT and of cerebral blood volume in pwMRI	• Black rating scale for shunt assessment: excellent, good, or fair	• Not reported	• 33% of patients had marked clinical improvement post-TT in the HHS and positive imaging (all shunted). 33%; TT negative or questionable but positive imaging (6 received a shunt). 33%; no clinical improvement nor positive imaging (not shunted) • At TT, there was a highly significant association between clinical assessment and perfusion changes (p < 0.01, w = 0.5) • No significant difference was found between HHS scores (7.94, positive group; 7.22, negative group) • No difference was found in CSF pressure values between positive and negative TT groups • TP = 8, FN = 5, FP = 1, TN = 1
Walter et al. (2005) [66]	n = 14	• Assessments: pre- and post-shunt: neurological exam, walking test, psychometric examination (MMS), HHS, and urinary incontinence protocol • STT involved 40–50 mL removal of CSF + MRI assessment • Only those who improve following STT (either clinically or increased perfusion in PWI-MRI) had shunt • Follow up: 6 months	• Minimum – 4 points on HHS scale • Walking test: step number reduction or 10% increase in average pace length	• Black rating scale for shunt assessment: excellent, good, or fair	• Not reported	• 25% of patients demonstrated increased cerebral perfusion and clinical improvement post-TT. 32% of patients demonstrated increased cerebral perfusion, but no significant change in clinical assessment. 46% of patients demonstrated neither a change in cerebral perfusion nor clinical assessment • 57% of patients had improved cerebral perfusion after STT • There was a significant difference between clinical assessment and perfusion changes (p < 0.01, effective size (w) = 0.63) • In the “improved” group, the pre-STT baseline perfusion values were significantly lower than the “no improvement” group. Significant increases from pre- to post-STT were only found in the “improved” group • STT: sensitivity = 50%, specificity = 50% • TP = 6, FN = 6, FP = 1, TN = 1
Ishikawa et al. (2012) [26]	n = 100	• Assessments: iNPHGS, MMSE, and the 3 m TUG. Post-shunt: mRS. Secondary outcome measures: iNPHGS, MMSE, and TUG as secondary outcome measures • TT involved ≥ 30 mL removal of CSF • Follow up: 3, 6, and 12 months	• iNPHGS: ≥ 1-point improvement • TUG: ≥ 10% improvement in time • MMSE: ≥ 3-point improvement	• mRS: 1 point or more improvement over 12 m	• Not reported	• CSF pressure at TT was significantly higher (p < 0.05) in shunt responders (median = 13 mmHg) than non-responders (median = 12 mmHg) • TT total (improvement in iNPHGS or MMSE or TUG): sensitivity = 92.5%, specificity = 20% • iNPHGS: total scale improvement: sensitivity = 71.3%, specificity = 65%. GS-gait change: sensitivity = 51.3%, specificity = 80.0%. GS-cognitive change: sensitivity = 25.0%, specificity = 85.0%. GS-urinary continence change: sensitivity = 37.5%, specificity = 85.0% • TUG: sensitivity = 34.2%, specificity = 73.6% • MMSE: sensitivity = 63.8%, specificity = 30% • GS total change and CSFP ≥ 15 cm H2O: sensitivity = 82.5%, specificity = 65.0% • TP = 74, FN = 6, FP = 16, TN = 4
Ishikawa et al. (2016) [27]	n = 61	• Assessments: TUG, 10Ti (10-m walk in time), and the 10St (10-m walk in steps) pre-TT and on days 1 and 4 post-TT • TT involved 30 mL of CSF drainage • Follow-up: 3 months	• iNPHGS: ≥ 1-point improvement or ≥ 10% improvement in TT quantitative measures	• ≥ 1-point improvement on the grading scale	• Not reported	• TUG day 1 post-TT: sensitivity = 78.3%, specificity = 80% TUG day 4 post-TT: sensitivity = 73.2%, specificity = 50.0% • 10Ti day 1 post-TT: sensitivity = 63.0%, specificity = 66.6% 10Ti day 4 post-TT: sensitivity = 63.4%, specificity = 90.0% • 10St day 1 post-TT: sensitivity = 41.3%, specificity = 86.7% 10St day 4 post-TT: sensitivity = 41.5%, specificity = 80.0% • TUG and 10Ti times significantly decreased (ANOVA: TUG p = 0.044; 10Ti p = 0.015) from pre-TT to day 1 post-TT and from pre-TT to day 4 post-TT

Studies included assessing the use of tap test (TT) in predicting shunt responsiveness. *SR*, shunt response; *TUG*, timed up and go test; *iNPH*, idiopathic normal pressure hydrocephalus; *mRS*, modified Rankin scale; *SPECT*, single-photon emission computerized tomography; *pwMRI*, perfusion-weighted imaging MRI; *HHS*, Homburg hydrocephalus scale; *STT*, spinal tap test; *iNPHGS*, iNPH grading scale; *10S*t, 10-m walk in step test; 10Ti, 10-m walk in time test.

### Gait tests

Three studies used gait function to assess TT response, namely the timed up and go (TUG) [26, 27, 71], 10-m walk in time (10Ti) [27], and in step (10St) tests [27]. Using the TUG test, Yamada et al. [73] showed that the absolute difference in time between pre- and post-TT was more accurate in predicting SR than percentage change. Using the absolute time difference, a ≥ 5.6-s threshold within 24 h after TT was reported to have a sensitivity = 83.3% and specificity = 81.0% for predicting an improvement of ≥ 10 s 12-month post-shunt [73]. Ishikawa et al. [26] set the threshold to a 10% improvement in the TUG, yielding sensitivity = 34.2% and specificity = 73.6%. Ishikawa et al. [27] again evaluated the TUG as well as 10Ti and 10St and found the percentage change in TUG on day 1 post-TT at the cut-off value ≥ 11.3% to have the highest sensitivity (78.3%) and specificity (80.0%).

### iNPH grading scales

Wikkelsø et al. [70] used an iNPH grading scale (iNPHGS) with 4 domains (gait, neuropsychology, balance, and continence). They found the TT to be limited in its ability to predict SR, with a sensitivity = 52% and specificity = 59% [70]. However, gait improvement with TT correlated significantly with improvement in post-shunt iNPH score (p = 0.02) [70]. Ishikawa et al. [26] utilized a slightly different iNPH grading scale with 3 domains (gait, cognition, and urinary function), but in line with Wikkelsø et al. [70], they also found low sensitivity (< 40%) and specificity (< 40%) values. However, these values improved slightly when the 3 domain scales were added as iNPHGS total change (1-point improvement in any domain), which had sensitivity = 71.3% and specificity = 65% [26]. Improvement in any of iNPHGS, TUG, or MMSE showed the highest sensitivity of 92.5%, but specificity was very low (20%) [26].

### Perfusion studies

In addition to clinical examinations, Hertel et al. [24] and Walter et al. [66] used magnetic resonance imaging (MRI) pre- and post-TT investigating cerebral perfusion and showed that TT diagnostic effectiveness can be improved by MRI use. Notably, Walter et al. [66] found that baseline perfusion values in the shunt responder group were significantly lower than in those who did not improve. Furthermore, they found that only the shunt responder group demonstrated a significant increase in cerebral perfusion from pre- to post-TT [66]. Both studies reported clinical examination to not be significantly associated with SR, unlike cerebral perfusion improvement [24, 64].

### Infusion test

Eight studies discussed the use of IT in predicting SR, analyzing different aspects of CSF dynamics (Table 3) [2, 4, 15, 29, 35, 39, 56, 59].

**Table 3:** Infusion test

Study	Shunted patients	Methodology	Criteria for + ve LIT	Criteria for SR	Side effects	Main reported outcomes
Meier and Miethke (2003) [41]	n = 155	• Assessments: formula for NPH recovery rate building upon Black grading scale for shunt assessment and Kiefer and Steudel’s clinical grading scale for NPH scores was used • Patients underwent intrathecal infusion tests with constant ICP monitoring • Follow up: 7 months	• Not reported	• Excellent, improved, and fair were classified as responders using their new model	• Not reported	• Overall, patients with Rout > 15 mm Hg/mL/min showed a significantly more favorable clinical course (p < 0.01) • In particular, the LIT predicts favorable outcomes for early-stage NPH patients with Rout > 15 mm Hg/mL/min; or in late-stage NPH patients (with cerebral atrophy) with Rout > 20 mm Hg/mL/min
Bech-Azeddine et al. (2005) [4]	n = 30	• Assessments: MMSE, global deterioration scale and ordinal scales of gait, incontinence, and cognition • LIT: Rout and ICP were monitored • Follow up: 1–3 months and 12 months	• Rout > 16.0 mmHg/mL/min	• 1 point was assigned for each degree of reduction or improvement in the scales of gait, incontinence, global deterioration scale and MMSE score (5-point change in MMSE = 1 degree change) • An NPH improvement was concluded, when a summation of the improvement and reduction scores was 2 or above	• Not reported	• 80% of the patients had a conclusive decision regarding shunting after LIT • 17 patients then underwent shunting, and the clinical improvement was 82%. However, including the patients undergoing an intraventricular assessment, the shunt success rate was 76% • TP = 9, FN = 2, FP = 2, TN = 2
Kahlon et al. (2005)[29]	n = 47	• Assessments: walk test, reaction time test, memory and identical forms test • LIT: Rout, PpL, and PPPA were measured • CSF pressure: recorded continuously for minimum 45 min to achieve the steady-state pressure plateau • Follow-up: 3–12 months	• Infusion pressure plateau level > 22 mmHg	• Significant improvement was a 5% (walk and reaction time) and 25% (memory and identical forms) increase relative to the best of baseline scored in the respective tests • Minimum 2 out of 4 different tests needed to show improvements relative to baseline in order to classify the patient as clinically improved	• In 1 patient the infusion was stopped < 45 min as pressure exceeded > 50 mmHg	• No significant differences between the levels of PpL and of Rout values between improved and non-improved patients were found. 78% of patients with PpL > 22 mmHg improved • PPPA (20 mmHg or above) correlated with Rout and PpL, but a high PPPA did not incur more improved patients than Rout and PpL • TP = 13, FN = 25, FP = 1, TN = 10
Sorteberg et al. (2004) [61]	n = 15	• Assessments: NPH score • Continuous computerized ICP monitoring for 24 h, then LIT using a 1.5 mL/min infusion rate, Rout measured • Follow-up: 6 months	• Not reported	• Post-shunt increases in NPH score relative to baseline	• No CSF leakage or complications were observed	• The NPH score was significantly correlated with a Rout of 12 mmHg/ml/min or above, pre- and post-shunting (r = 0.71, p = 0.005); gait: r = 0.81, p < 0.0001; incontinence: r = 0.81, p < 0.0001; dementia: r = 0.64, p = 0.014 • Radiological signs: absolute changes in third ventricle index, cella media index, and ventricular score were insignificantly correlated with Rout
Eide and Brean (2010) [15]	n = 45	• Assessments: NPH score • ICP monitoring for 8 h, and subsequently LIT using a 1.5 mL/min infusion rate, Rout measured • Follow-up: after 12 months	• Rout > 12.0 mmHg/mL/min	• Increase of ≥ 2 scores in NPH scale	• No complications	• 98% of shunted patients had Rout ≥ 12 mmHg/mL/min, of which 79.5% were shunt responders • Significant correlation between Rout and change in NPH score 12-month post-shunt was found (Spearman correlation 0.31, p < 0.04) • Significant correlation between change in NPH score after 12 months and elevated CSF pulse pressure (> 4 mmHg) measured during lumbar infusion was found (Spearman correlation 0.47; p = 0.002). Sensitivity = 88%, specificity = 60%, PPV = 89%, NPV = 60% • TP = 35, FN = 0, FP = 9, TN = 1
Anile et al. (2010) [2]	n = 96	• Assessments: Stein-Langfitt and Larrson scores • 1 mil/min 30-min VIT, measuring Rout and IE • Patient split into VIT showing an IE slope of < 0.25 IE, > 0.25 IE, and > 0.30 IE • Some patients were shunted without VIT • Follow-up: 6–12 months	• Not reported	• Minimum + 1 point in the Stein-Langfitt score and + 2 points in the Larsson score; evaluated by the clinician and the patient’s relatives	• Not reported	• No significant relationship between Rout and clinical improvement was found • But an IE slope value of 0.25 clearly differentiated between the improved and unimproved patients: the mean IE slope was 0.49 (range 0.34–0.76) in shunt responders and 0.19 (range 0.13–0.25) in non-responder (p < 0.00001) • In the VIT groups, 95% and 100% at cut-off > 0.25 IE and > 0.30 IE, respectively, improved post-shunt. In the non-VIT group, only 69% improved
Mahr et al. (2016) [37]	n = 31	• Assessments: Kiefer score, SLHS, MMSE, standardized gait testing, grooved pegboard test, and mRS • Overnight ICP monitoring for 24 to 48 h, LIFT, measuring Rout and ELD were performed • Follow-up: 12 months	• Not reported	• Positive change in Kiefer score values 12 months postoperatively relative to preoperative baseline score	• None reported	• Rout values were significantly different between ELD-non-responders (mean = 18 mmHg/mL/min) and ELD-responding patients (< 12 mmHg/mL/min) • High Rout patients showed less post-shunting improvement than patients with low/normal Rout values (p = 0.039) • Rout cut-off at ≥ 12 mmHg/mL/min: sensitivity = 79%; specificity = 51%, PPV = 53%, NPV = 78% • Optimum Rout cut-off based on Youden Index = 13 mmHg/mL/min • TP = 8, FN = 2, FP = 7, TN = 8
Ryding et al. (2018) [58]	n = 31	• Assessments: walk test (walk at maximum speed for 18 m, mean velocity is calculated) • LIT: plateau pressure (baseline + half pulse amplitude), baseline ICP, Max increase in intracranial CSF volume of Vin(max), volume at plateau level of intracranial venous blood volume reduced after CSF infusion (PLIV) and pulse wave • Follow-up: 2–22 months, except one subject 10 years later	• Max. LIT plateau pressure ≥ 22 mmHg	• Minimum ≥ 20% postoperative walk speed increase relative to preoperative baseline	• None reported	• LIT plateau pressure and Vin(max) pressure did not significantly predict shunt responsiveness • PLIV of < 14 mL was positively predictive of shunt responsiveness (p = 0.01) • TP = 21, FN = 0, FP = 1, TN = 4

Studies included assessing the use of infusion test (IT) in predicting shunt responsiveness. *SR*, shunt response; *NPH*, normal pressure hydrocephalus; *LIT*, lumbar infusion test; *Rout*, resistance to outflow; *MMSE*, mini mental state examination; *PpL*, steady-state plateau pressure; *PPPA*, plateau pulse pressure amplitudes; *ICP*, intracranial pressure; *VIT*, ventricular infusion test; *IE*, intracranial elastance; *Vin(max)*, maximal increase in intracranial CSF volume; *PLIV*, volume at plateau level of the part of the intracranial venous blood volume that can be decreased due to infusion of CSF.

### Rout

Seven studies analyzed the role of Rout, defined as resistance to outflow of CSF which reflects the impedance of CSF absorption [2, 4, 15, 29, 35, 39, 59]. Five studies concluded that Rout significantly predicts SR; however, there were discrepancies in the recommended Rout cut-off value [4, 15, 35, 39, 59]. While Meier and Miethke [41] and Bech-Azeddine et al. [4] reported significant cut-off values at 15 mmHg/mL/min and 16 mmHg/mL/min, respectively, Mahr et al. [37] reported the optimal Rout cut-off to be at 13 mmHg/mL/min. However, Kahlon et al. [29] and Anile et al. [2] found no significant correlation between Rout and SR.

### Complementary variables

Besides Rout, the significance of complementary variables was reported by 4 studies [2, 15, 29, 56]. Kahlon et al. [29] concluded that steady-state plateau pressure (PpL) was equal to Rout in terms of predicting SR, with 78% of patients with PpL > 22 mmHg improving. Eide and Brean [15] detected a significant correlation between 12-month shunt outcome and elevated CSF pulse pressure (CSFPP) amplitude of ≥ 4 mmHg measured during lumbar infusion (p < 0.002). Anile et al. [2] found that a cut-off > 0.25 for intracranial elastance (IE) slope gradient clearly differentiated shunt responders and non-responders and was superior to Rout in predicting SR in ventricular infusion test (VIT). Ryding et al. [58] concluded that the volume at plateau level of intracranial venous blood volume (PLIV) decreasing after CSF infusion was positively predictive of shunt outcomes (p < 0.01).

### Extended lumbar drainage

Eight studies used an ELD for the prediction of SR using different response criteria for ELD (Table 4) [7, 8, 17, 19, 35, 36, 47, 69].

**Table 4:** Extended lumbar drainage

Study	Shunted patients	Methodology	Criteria for + ve ELD	Criteria for SR	Side effects	Main reported outcomes
Gallina et al. (2018) [19]	n = 68	• Assessments: MMSE, objective urinary incontinence and gait scale (4 categories from functional to completely dysfunctional) • 24-h ELD • Follow-up: 12 months	• + 2 points (urinary incontinence + gait scale) or + 1 on urinary incontinence scale or gait scale and minimum 3 points on MMSE)	• + 2 points (urinary incontinence + gait scale) or + 1 on urinary incontinence scale or gait scale and minimum 3 points on MMSE)	• Intracranial hypotension (n = 1), root irritation (n = 2), headache (9.9%), overall procedural complications (2.1%)	• 73.3% of patients had a positive outcome to 1-day ELD • ELD had a sensitivity = 100%, specificity = 75.0%, PPV = 96.8%, NPV = 100% • TP = 60, FN = 0 FP = 2, TN = 6
Marmarou et al. (2005)[38]	n = 102	• Assessments: MMSE, gait and bladder function, and neuro-psychometric parameters • 3-day ELD • A shunt was offered based on ELD response or patient request • Follow-up: 12 months	• A 10-day survey by patients and caregivers stating improvement in clinical status	• The same criteria used to assess 3-day ELD response were used to assess surgical outcome	• Infection in 2 (1.3%) of 151 patients, and 4 patients (2.6%) experienced headache	• There was a statistically significant correlation between ELD responders and shunt responsiveness (p < 0.0001) • ELD prediction of shunt responsiveness has a sensitivity of 95% (CI: 84–90%), specificity of 64% (CI: 44–84%), PPV of 90% (CI: 72–100%), NPV of 78% (CI: 70–80%) • TP = 76, FN = 4, FP = 8, TN = 14
Chotai et al. (2014) [8]	n = 60	• Assessments: videography of gait assessment, balance, muscle strength, speech fluency, behavior, and MMSE • A 4-day ELD • Follow-up: 12 months	• Increase of ≥ 2 points on MMSE or improvement in gait, balance speech fluency was assessed based on videotape assessment and documentation	• Gait and cognitive improvement (MMSE improvement of 2 >) were the primary outcomes • Functional outcomes were assessed using a survey by the family and patient	• 10% of ELD patients experienced. Transient nerve root irritation	• A statistically significant improvement in cognition on day 4 following ELD was observed with median MMSE score increasing to 27 from 23.5 (χ2 = 15.74, p = 0.001), with no improvement in gait • 4-day ELD: sensitivity = 100%, specificity = 60%, NPV = 100%, and PPV = 96% • ROC analysis demonstrated reasonable accuracy for ELD prediction of SR (area under curve = 0.8 ± 0.14, p = 0.02, CI = 0.52–1.0) • TP = 55, FN = 0, FP = 2, TN = 3
Mahr et al. (2016) [37]	n = 31	• Assessments: Kiefer score, SLHS, MMSE, mRS standardized gait testing, and grooved pegboard test • A 3-day ELD • Follow-up: 12 months	• A 10% improvement in gait or a 10% improvement in MMSE • Qualitative assessment by patient and family also considered	• A ∆Kiefer values 12-month post-shunt was used to assess SR with patients categorized as excellent responders (relief of all symptoms), improved patients (∆Kiefer reduction of at least 10%), and non-responders (∆Kiefer reduction < 10%)	• Not reported	• ELD response predicted improvement post-shunt surgery in 87.9% of patients with iNPH
Eide and Stanisic (2010) [17]	n = 31	• Assessments: gait analysis and NPH scale • 3-day ELD was used with ICPM • Follow-up: 3 and 6–12 months	• Gait function was assessed using a video analysis with an improvement in gait function as a positive ELD response	• Increase + ≥ 2 scores on their NPH scale	• Not reported	• ELD responders and non-responders had significantly different ICP wave amplitudes (p < 0.001) • All patients (53.6%) with elevated pulsative ICP had a clinical response to ELD, compared to 23.1% of the low ICP group (PPV = 100% and NPV = 77% for clinical response to ELD) • The reduction in ICP wave amplitude during ELD was related to the changes in NPH scores (Spearman correlation − 0.6; p < 0.001) after shunt treatment • TP = 15, FN = 1, FP = 3, TN = 2
Panagiotopoulos et al. (2005) [47]	n = 22	• Assessments: history taking, neurological exam, MMT and NPH score • 5-day ELD • Follow-up: 3 month	• + 1 or more points on the NPH scale	• Increase in NPH score	• None reported	• In patients able to walk, improvement after ELD in gait disturbance was significantly correlated with improvement 3-month post-shunting (Pearson’s r = 0.833, p < 0.01) • Quantitative NPH score analysis for 3-month post-shunt correlated to an improvement after ELD (Spearman’s rho = 0.462, p = 0.03) • TP = 9, FN = 2, FP = 0, TN = 11
Chaudhry et al. (2007) [7]	n = 60	• Assessments: RAVLT, Boston naming test, COWA, Wechsler memory logical memory test, alphabet writing, line tracing and coping pentagons, Rey complex figure test and grooved pegboard test • A 2–3-day ELD • Follow-up: 3 months	• 1 SD or more improvement in RAVLT/WMS	• 1 SD or more improvement in RAVLT/WMS	• None reported	• In 3 subsets evaluating learning, retention and delayed recall of the RAVLT, the magnitude of improvement post-ELD insertion was predictive of the magnitude of improvement after shunt surgery: learning (r ^2^ = 0.58; p < 0.001), retention (r^2^ = 0.32; p = 0.04), and delayed recall (r ^2^ = 0.36; p = 0.02) • A 5- or more point improvement on RAVLT post-drainage was associated with a significant improvement on > 0.5 the memory tests post-VPS (chi-squared = 10.8; p = 0.0005) and had PPV = 50% and NPV = 96%
Woodworth et al. (2009) [71]	n = 51	• Assessments: objective symptom analysis • Controlled continuous CSF drainage at 10 mL/h (240 mL/day) for 3 days • Follow-up: 1, 3, 6, 12 months and yearly thereafter	• ≥ 1 iNPH symptom improvement • OR probable B-waves present during Pcsf monitoring	• Objective and family reported improvement in 1, 2, or 3 iNPH symptoms	• None reported	• Continuous lumbar drainage prediction of SR: sensitivity = 91%, specificity = 70% • Patients with a positive response to CSF drainage were 3.2 times more likely to improve following CSF shunting (RR = 3.2; [CI: 0.09–1.00], p < 0.05) • A positive CSF drainage response predicted VPS responsiveness (RR = 0.30, [CI: 0.09–0.98], p < 0.05)

Studies included assessing the use of extended lumbar drainage (ELD) in predicting shunt responsiveness.

*COWA*, controlled oral word association test; *CSF*, cerebrospinal fluid; *ICP*, intracranial pressure; *iNPH*, idiopathic NPH; *MMT*; mini mental test; *MMSE*, mini mental state exam; *mRS*, modified Rankin scale; *NPH*, normal pressure hydrocephalus; *Pcsf*, CSF pressure; *TT*, tap test; *VPS*, ventriculoperitoneal shunt; *RAVLT*, Rey auditory visual learning test; *SD*, standard deviation; *SLHS*, Stein and Langfitt hydrocephalus score; *SR*, shunt response; *WMS*, Wechsler memory scale.

### ICPM as assessment of ELD response

Eide and Stanisic [17] reported a correlation of ICPM with ELD, showing that all patients with a raised pulsatile ICP of ≥ 5 mmHg in 10% of recording time were ELD responders (PPV = 100% and NPV = 77%). A reduction in ICP wave amplitude during ELD was a powerful indicator of SR (Spearman correlation − 0.6; p < 0.001) [17].

### Memory tests as criteria for ELD response

Gallina et al. [19] showed that a 1-day ELD had a sensitivity = 100% and specificity = 75% for SR when using a ≥ 3-point increase in MMSE for ELD response. This was supported by Chotai et al. [8] who used a 4-day ELD with ≥ 2-point MMSE increase, yielding sensitivity = 100% and specificity = 60%. Similarly, Mahr et al. [37] found that an ELD response, defined as a 10% improvement in gait or MMSE score, was predictive of SR in 87.9% of patients. Using a different memory test, Chaudhry et al. [7] assessed ELD response with Rey auditory verbal learning test (RAVLT), with improvement post-ELD yielding PPV = 50% and NPV = 96%.

### Global symptom improvement as criterion for ELD response

Panagiotopoulos et al. [47] found that a ≥ 1-point increase in NPH score, an objective assessment of symptom severity, after ELD was significantly associated with SR. This finding was supported by Woodworth et al. [71] who reported that ≥ 1 objective symptom improvement predicted SR with sensitivity = 91%, and specificity = 70%. Marmarou et al. [38] showed that when improvement was reported by the patient or family, ELD response was significantly correlated to SR (p < 0.001) and had sensitivity = 95% and specificity 64%.

### Intracranial pressure monitoring

Eleven studies investigated the use of ICPM parameters for predicting SR (Table 5) [12–14, 16–18, 20, 35, 49, 59, 60].

**Table 5:** Intracranial pressure monitoring

Study	Shunted patients	Methodology	Criteria for SR	Side effects	Main reported outcomes
Stephensen et al. (2005) [62]	n = 13	• Assessments: gait, balance, social function, climbing stairs, psychometric test • Overnight intraparenchymal ICPM (17 to 26 h) at 50 Hz (B-wave analysis) 1-day pre-shunting • Follow-up: 3–6 months	• Mean of difference between results from preoperative and postoperative test battery, > 0 implies improvement	• Not reported	• B-waves were seen in all NPH patients • No significant correlation between percentage of B-waves and post-shunting outcomes were found
Mahr et al. (2016) [37]	n = 31	• Assessments: Kiefer score, SLHS, MMSE, standardized gait testing, grooved pegboard test, and mRS • Overnight intraparenchymal ICPM was used for 24–48 h • Follow-up: 12 months	• Excellent (relief of all symptoms) • Improved (Kiefer score reduction of at least 10%)	• 1 patient experienced temporary neurological deficit, 2 patients were non-compliant	• RAP index, mean ICP, and slow wave amplitude did not differ significantly between shunt responders and non-responders • RAP > 0.8 shunt outcome prediction: sensitivity = 74%, specificity = 70%; PPV = 61%, NPV = 81% • RAP < 0.7 shunt outcome prediction: sensitivity = 91%, specificity = 41%; PPV = 55%, NPV = 86% • Slow wave > 1.5 shunt outcome prediction: sensitivity = 35%, specificity = 71%; PPV = 80%, NPV = 25% • TP = 9, FN = 3, FP = 6, TN = 14
Garcia-Armengol et al. (2016) [20]	n = 89	• Assessments: NPH score, MRI (DESH) • Overnight intraparenchymal ICPM (10 h) 1-day pre-shunting • Follow-up: 12 months	• Improvement in NPH score	• Not reported	• High ICP pulse amplitude (> 4 mmHg) was significantly more prevalent among shunt responders (84.4%) than non-responders (12%), p < 0.001 • ICP pulse amplitude > 4 mmHg: Youden index = 0.72, PPV = 94.7% and NPV = 68.8% • ICP pulse amplitude was more sensitive (84.4% vs 79.7%) and more specific (88.0% vs 80.0%) than a positive DESH finding in predicting SR • TP = 54, FN = 10, FP = 3, TN = 22
Sorteberg et al. (2004) [61]	n = 15	• Assessments: NPH score (including gait disturbance, urinary incontinence, and dementia) • Intraparenchymal ICPM was used for 24 h • Follow-up: 6 months	• Post-shunt increases in NPH score relative to baseline	• Not reported	• No relationship between the number of ICP elevations to 20 mmHg (lasting 0.5 or 1 min) and SR • No significant relationship between mean ICP and SR
Eide et al. (2010) [18]	n = 27	• Assessments: NPH grading scale (gait disturbance, urinary incontinence, and dementia) • Intraparenchymal ICPM and ABP monitoring • Software computed mean ABP, mean ICP, mean ABP wave amplitude, mean ICP wave amplitude, and cerebral perfusion pressure (CPP) measured in 6-s time windows • Shunt insertion 1–3-week post-assessment • Follow-up: 3, 6, 12 months	• Increase ≥ 2 scores on NPH scale	• Not reported	• Mean ICP wave amplitude was significantly increased in shunt responders compared with non-responders (p < 0.001) • Compared to other parameters (static ABP, static ICP), mean ICP wave amplitude (≥ 4 mm Hg) was highly predictive for SR (PPV = 100% and NPV = 100%) • TP = 21, FN = 0, FP = 0, TN = 6
Eide and Stanisic (2010) [17]	n = 31	• Assessments: NPH grading score • Continuous overnight intraparenchymal ICPM • Criteria for increased intracranial pulsatility: mean ICP wave amplitude ≥ 4 mm Hg on average in addition to mean ICP wave amplitude ≥ 5 mmHg in 10% of recording time • Follow-up: 3 and 6–12 months	• Increase of ≥ 2 scores on the NPH scale	• Not reported	• 95.8% patients with high pulsatile ICP were shunt responsive • Pulsatile ICP was significantly higher (p < 0.001) in shunt responders than non-responders • TP = 23, FN = 1, FP = 1, TN = 6 • Pulsatile ICP (mean ICP wave amplitude) was significantly correlated with NPH score (Spearman correlation − 0.47; p = 0.002) • Only 14.3% of patients with low pulsatile ICP were shunt responsive • ICP pulsatility: PPV = 96% and NPV = 86%
Pfisterer et al. (2007) [51]	n = 55	• Assessments: Dutch classification • Invasive CIPM was used for 48 h • ICP abnormally high: continuously > 10 mmHg • Positive ICPM: B-waves between 5 and 10% • Follow-up: 1 to 10 years (median 6.5 years)	• Improvement in gait/cognition/urinary incontinence on an ordinary scale using the Dutch classification	• 1 patient suffered from acute ventriculitis	• Positive CIPM followed by shunt insertion correlated with a significant improvement of gait (96.1%), memory (77.1%), and urinary disturbance (75.7%) (p < 0.004) • Patients with pressure levels > 10 mmHg improved following shunting (p < 0.01) • No significant relationship between B-wave amplitude and SR
Eide (2005) [13]	n = 39	• Assessments: NPH grading scale • Continuous intraparenchymal ICPM • The percentage time the mean ICP wave amplitude was ≥ 2 mmHg, ≥ 3 mmHg, ≥ 4 mmHg, ≥ 5 mmHg, ≥ 6 mmHg, or ≥ 7 mmHg was recorded within cconsecutive 6-s time windows during a 10-h recording • Follow up: 12 months	• An increase of ≥ 1 point in NPH score	• Not reported	• Mean ICP or ICP wave latency did not differ between shunt responders and non-responders • Mean ICP wave amplitude was significantly different between the groups (p < 0.001) • Mean ICP wave amplitude was significantly higher (p < 0.001) in those with a ≥ 1-point change in NPH score compared to those who did not • Mean ICP wave amplitude of ≥ 4 mmHg in 70% of time windows: PPV = 90% and NPV = 100% • Mean ICP wave amplitude of ≥ 5 mmHg in 40% of time windows: PPV = 89% and NPV = 91% • TP = 32, FN = 0, FP = 1, TN = 16
Eide and Brean (2006) [14]	n = 23	• Assessments: NPH grading scale • Intraparenchymal ICP monitoring • Elevated ICP amplitudes: when mean wave amplitudes were either ≥ 4 mmHg in ≥ 70%, ≥ 5 mmHg in ≥ 40%, or ≥ 6 mmHg in ≥ 10% of the recording time • Follow up: 12 months	• Increase in NPH score	• Minor complications in 4 patients (6.5%)—subcutaneous wound infections (treated with antibiotics)	• 91% of patients with elevated mean wave amplitudes (> 2 mmHg) demonstrated a marked improvement (median change in NPH score + 4) • Ranges of SR prediction of the threshold values: PPV = 82–90% and NPV = 91–100%
Eide (2011) [12]	n = 22	• Assessments: NPH grading scale • Overnight intraparenchymal ICPM, CO, and ABP wave amplitude monitoring • Elevated ICP wave amplitudes: average of mean ICP wave amplitude ≥ 4 mmHg in addition to mean ICP wave amplitude ≥ 5 mmHg in ≥ 10% of time recording • Follow up: 12 months	• An increase ≥ 2 in NPH score	• Not reported	• NPH score did not correlate to the CO and to ABP wave amplitude but correlated significantly to ICP wave amplitude (p = 0.003) • Patients with higher preoperative ICP wave amplitude levels showed greater improvement in iNPH symptoms at 12-month follow-up • Elevated ICP wave amplitude: sensitivity = 100%, specificity = 50% • TP = 16, FN = 0, FP = 3, TN = 13
Eide and Sorteberg (2010) [16]	n = 131	• Assessments: NPH grading scale • Intraparenchymal continuous ICPM • Abnormal intracranial pulsatility: average mean ICP of > 4 mmHg in addition to mean ICP wave amplitude of > 5 mmHg in > 10% of time recordings, in 6-s time windows • Follow-up: from 3 months (2-year median)	• An increase ≥ 2 in NPH score	• Not reported	• Threshold of mean ICP (8 mmHg): sensitivity = 51%, specificity = 74%, PPV = 88%, and NPV = 28% • Mean ICP wave amplitude ≥ 4 mmHg: sensitivity = 98%, specificity = 70%, PPV = 93%, and NPV = 91% • Mean ICP wave rise time coefficient threshold of 20 mmHg/s: sensitivity = 74%, specificity = 74%, PPV = 92%, and NPV = 43% • RAP threshold of 0.8: sensitivity = 66%, specificity = 48%, PPV = 82%, and NPV = 27% • TP = 100, FN = 2, FP = 8, TN = 20

Studies included assessing the use of intracranial pressure monitoring (ICPM) in predicting shunt responsiveness. *CT*, computerized tomography; *MRI*, magnetic resonance imaging; *ICP*, intracranial pressure; *ABP*, arterial blood pressure; *ICPM*, ICP monitoring; *SLHS*, Stein and Langfitt hydrocephalus score; *MMSE*, mini mental state exam; *mRS*, modified Rankin scale; *LIFT*, lumbar infusion test; *CIPM*, continuous ICPM; *ELD*, extended lumbar drainage; *RAP*, correlation coefficient between pulse amplitude and ICP; *DESH*, disproportionately enlarged subarachnoid space hydrocephalus; *NPH*, normal pressure hydrocephalus; *INPH*, idiopathic NPH.

### Static ICPM

Six studies evaluated the use of the static ICP parameter (mean ICP and B-wave activity) as a predictor of SR [13, 16, 35, 49, 59, 60]. Overall, 4 studies reported no significant correlation between mean ICP or B-wave activity and SR [13, 35, 59, 60]. However, Pfisterer et al. [51] found a significant correlation (p < 0.004) between patients with a preoperative basal ICP level > 10 mmHg and SR. Eide and Sorteberg [16] found the mean ICP to be significantly higher in shunt responders (8 mmHg vs. 7 mmHg), although static ICP overall was a poor predictor of SR.

### Dynamic ICPM

Seven studies reported a significant correlation of dynamic ICP values with SR [12–14, 16–18, 20]. Eide and Brean [14] examined pulse amplitude value (PAV) cut-offs of ≥ 4, ≥ 5, and ≥ 6 mmHg in 70%, 40%, and 10% of 6-s time windows, and reported a PPV = 82–90% and NPV = 91–100%. It was also reported previously by Eide [13] that a PAV ≥ 4 mmHg in 70% of the recording time was a significant predictor of SR, PPV = 90% and NPV = 100%. Eide and Sorteberg [16] also reported that a mean ICP wave amplitude cut-off of ≥ 4 mmHg had sensitivity = 98% and specificity = 70%, PPV = 93%, and NPV = 91%. Eide (2005) [13], Eide et al. [18], and Eide (2011) [12] additionally found that ICP wave amplitude values did not correlate with cardiac output (CO) or arterial blood pressure (ABP) wave amplitude increases.

### RAP index

The relationship between static and dynamic ICP parameters was measured in 2 studies using the RAP index (Pearson correlation between mean ICP and mean ICP wave amplitude) [16, 35]. Eide and Sorteberg [16] reported that although the RAP average was similar between responders and non-responders, the percentage of RAP ≥ 0.6 or ≥ 0.8 was higher in shunt responders. The use of a RAP threshold of 0.8 yielded sensitivity = 66%, specificity = 48%, PPV = 82%, and NPV = 27% for predicting SR [16]. In line with this, Mahr et al. [37] reported sensitivity = 74% and specificity = 70% for RAP ≥ 0.8.

### Meta-analysis

In the meta-analysis, sensitivity and specificity of the best-performing diagnostic parameters were selected for each included study to facilitate consistent comparisons. Studies were excluded from the meta-analysis in the case of insufficient reporting of statistical findings. A meta-analysis was not conducted for the diagnostic ability of presenting features due to significant methodological heterogeneity.

**Fig. 3:**
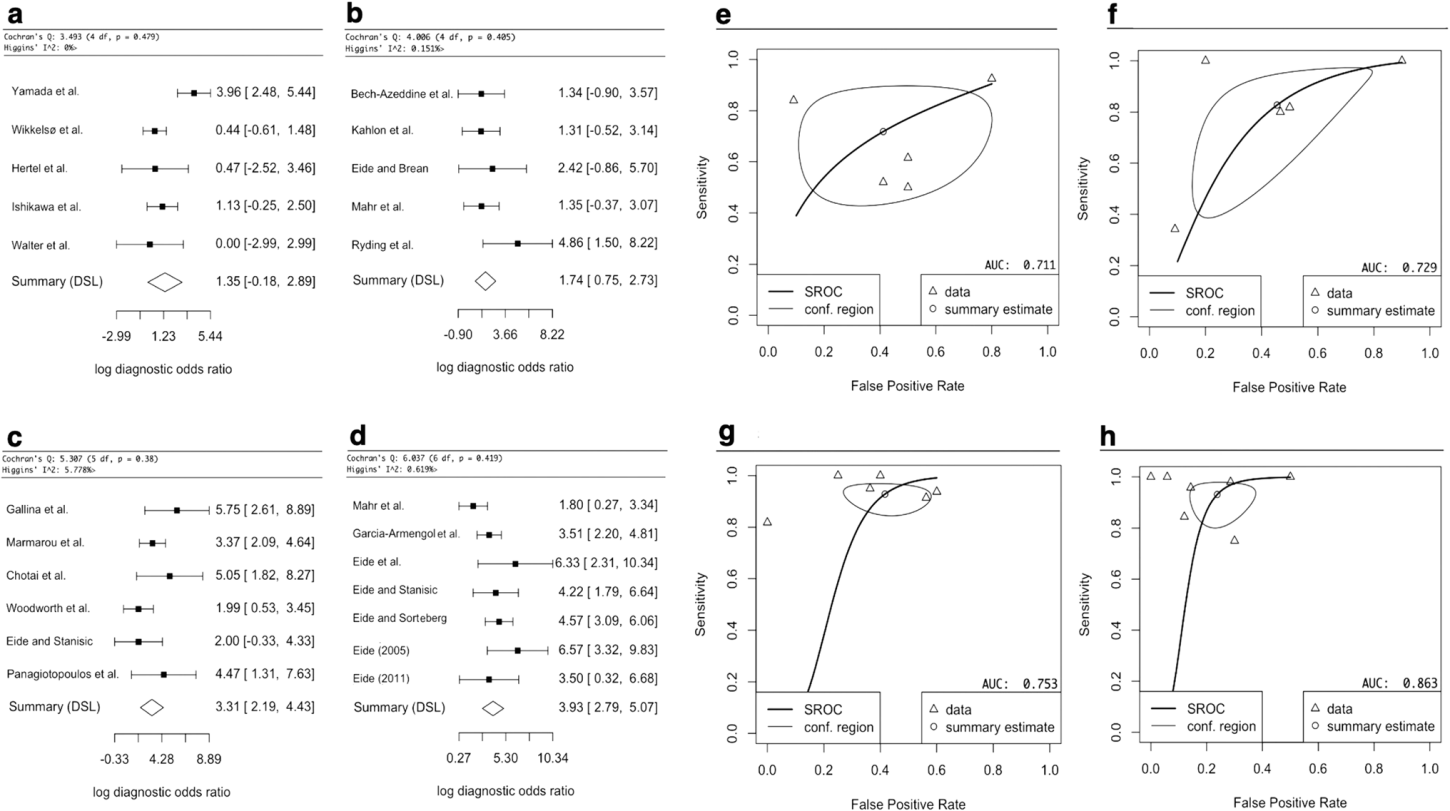
Forest plots indicating the log diagnostic odds ratio (logDOR) of TT (tap test) in Fig. 3a, IT (infusion test) in Fig. 3b, ELD (extended lumbar drainage) in Fig. 3c, and ICPM (intracranial pressure monitoring) in Fig. 3d, as well 95% confidence intervals in brackets. At the bottom of each graph, the DerSimonian-Laird (DSL) summary point is shown, which is the summary diagnostic odd ratio (DOR), drawn as a diamond with width inversely proportional to its standard error. A higher DOR implies higher diagnostic effectiveness. Cochrane Q test [9] is shown with p < 0.05% indicating significance, and Higgin’s I^2^ test [68] with < 25%, 25–50%, > 50% indicating low, moderate, and high heterogeneity, respectively. Study-specific estimates of sensitivity and false-positive rate values are shown in summary receiver operating characteristics (SROC) curve for TT in Fig. 3e, IT in Fig. 3f, ELD in Fig. 3 g, and ICPM in Fig. 3 h. The SROC curve is drawn in black, and the confidence region (95%) is drawn in grey. The data points are symbolized by triangles, and the summary estimate as a circle. The area under the curve (AUC) is shown in the bottom right corner with an AUC of 1 indicating highest diagnostic effectiveness and an AUC of 0.5 or below indicating an ineffective test

The meta-analysis for TT included 5 studies, and the DSL summary point is at 1.35 (95% CI: − 0.18–2.89) (Fig. 3a, Fig. 3e) [24, 26, 64, 68, 71]. The analysis of IT included 5 studies, and the DSL summary point is at 1.74 (95% CI: 0.75–2.73) (Fig. 3b, Fig. 3f) [4, 15, 29, 35, 56]. For ELD, 6 studies were included, and the DSL summary point is at 3.31 (95% CI: 2.19–4.43) (Fig. 3c, Fig. 3g) [8, 17, 19, 36, 47, 69]. Finally, for ICPM, 7 studies were included, and the DSL summary point is at 3.93 (95% CI: 2.79–5.07) (Fig. 3d, Fig. 3h) [12, 13, 16–18, 20, 35]. Cochrane’s Q test [9] and Higgins’ I^2^ test [68] indicate low heterogeneity for all sub-analyses (Fig. 3).

Overall, ICPM had the highest DOR (50.9) and AUC (0.877), followed by ELD (DOR = 27.7, AUC = 0.753), then IT (DOR = 5.70, AUC = 0.729), and finally TT (DOR = 3.86, AUC = 0.711).

## Discussion

Correctly identifying suitable patients for shunt surgery remains challenging but crucial, as unlike secondary NPH, a large proportion of iNPH patients have poor shunt outcomes. Given that both up to 41% of iNPH patients do not have a positive SR [23], as well as shunting carrying a significant procedural risk of 22–28% [31, 70], the need for an accurate diagnostic test to predict SR is apparent. Hence, this review offers guidance as to which clinical tests and patient factors are most effective in predicting SR.

Our meta-analysis of 21 studies concluded that ICPM is the most effective clinical predictor of SR, followed by ELD, IT, and TT. The findings suggest that a patient with shunt-responsive iNPH is 50.1 × more likely to have positive ICPM than a shunt-unresponsive iNPH patient, compared to 27.7 × more likely with ELD. In comparison, a patient with shunt-responsive iNPH is only 5.7 × more likely to have a positive IT and only 3.86 × more likely to have positive TT.

The most predictive indicator of SR is a mean pulse amplitude cut-off of ≥ 4 mmHg, in 70% of recording times in dynamic ICPM [17, 19, 29]. The meta-analysis is a reliable indicator of ICPM effectiveness as the methodology of the studies included was similar, all using intraparenchymal ICP monitors and assessing similar index parameters, reflected by the relatively small spread of data (Fig. 3h). However, we found several limitations within the included studies. In papers with Eide as principal author, which comprised 5 of the 7 included ICPM studies [12, 13, 16–18], there were notable differences in patient selection, with Sorteberg et al. [61] selecting patients with triad symptoms and ventriculomegaly for ICPM, while Eide and Stanisic [17] and others used the Relkin guidelines (Table 5) [56]. Moreover, 4 of these studies selected patients retrospectively from the same unit between 2002 and 2008, raising concerns about patient selection bias; additionally some patients may have been excluded from retrospective analysis in one study but included in another [13, 16–18]. Ultimately, further research is needed in multicenter settings, with more attention on complications, which Eide neglected. Overall, given the limitations of the included ICPM studies, the findings of the meta-analysis for ICPM must be interpreted with caution. Although TT is thought to simulate a shunt procedure making it an accurate assessment of the effect of a physiological reduction of CSF circulating volume, its high false-negative rates may deprive many patients of the potential benefits of shunt surgery. ICPM could help identify these patients when used as a second-line test given its higher specificity. The mechanism of ICPM’s greater accuracy may be due to its ability to uncover CSF pathology, which affects the compliance of brain parenchyma, by direct analysis of its reflection in ICP values. However, as the pathophysiology of iNPH remains unknown [44], the biological mechanism behind the statistical diagnostic superiority of ICPM remains elusive. Regardless of this, a significant advantage of ICPM is that it utilizes objective cut-off values based on monitor readings, and, therefore, is not subject to assessor bias like in some subjective assessments of ELD or TT improvement. ICPM is negatively regarded due to a high perceived risk of complications. However, it must be noted that intraparenchymal ICPM has been found to be much safer than external ventricular drain (EVD)-ICPM: Raboel et al. [54] reported a 5% infection risk for intraparenchymal ICPM versus 27% for EVD-ICPM [22]. In line with this, Vonhoff et al. [64] reported no patients with major complications and only 7% with minor complications, such as accidental removal of the probe, in 152 adults undergoing hydrocephalus ICPM. Although currently less accurate than invasive ICPM, non-invasive ICPM, using transcranial Doppler ultrasonography or MRI, might provide an entirely complication-free assessment of SR in the future [22, 54].

ELD is the second most effective test. Despite ELD techniques and response criteria varying significantly between studies, there was relatively low spread in the data (Fig. 3c, Fig. 3g), indicating that ELD is highly accurate regardless of methodological heterogeneity. That said, 3-point MMSE improvement after 1-day ELD had the highest sensitivity and specificity and hence should be given preference [19]. Furthermore, as Chotai et al. [8] found that 1-day ELD is as effective as 4-day ELD, a 1-day ELD should be used to avoid procedural complications and financial costs of a 4-day ELD. However, it must be noted that the study by Chotai et al. [8] had several weaknesses, particularly supplementary tests, such as radionucleotide cisternography and MRI, being used, which hinders drawing valid conclusions regarding ELD as a sole diagnostic test for SR. Interestingly, the Japanese NPH guidelines [44] recommend TT over ELD, citing fewer complications in the TT, while still reporting higher sensitivity and specificity of ELD. However, we only found 3 cases of infections and no other long-term significant complications in 8 ELD studies (n = 425), consistent with Walchenbach et al. [65] who reported only 2 major complications (n = 49) without long-term sequelae. We conclude that the much higher diagnostic effectiveness of ELD relative to TT and IT outweighs the potential complication risk, which has been decreasing in the last decade most likely due to evolving ELD techniques [8].

The financial burden of ICPM and ELD is regarded by many as significant obstacle to clinical implementation. In 2006, Burnett et al. [6] found that an undiagnosed NPH patient would incur mean costs of $108,842. In comparison, it costs $7000 to insert a shunt and $2870 for an ELD, both values signifying the cost of the respective devices only [6]. However, given the low NPV and specificity of ELD, it may incur additional costs by not selecting shunt-responsive patients (Table 4). This could be avoided by using a highly specific diagnostic test, namely ICPM; however, the same authors reported that a hypothetical diagnostic test with specificity = 80% would incur $83,000 per quality-adjusted life year, due to the complexity of prolonged clinical monitoring which may exceed cost-effectiveness thresholds [6]. That said, since 2006, many studies reported ICPM and ELD specificity to be > 80% [13, 15, 17, 20, 47]; hence, we recommend an update on the current cost-effectiveness of both tests.

Given the high accessibility of IT and TT, they can be used as first-line diagnostic procedures. Patients who test negatively, but have a high index of clinical suspicion, should be followed up with ICPM, or alternatively with ELD, as these tests have significantly higher specificity and sensitivity. For IT, we recommend using Rout with a cut-off range between 13 and 16 mmHg [4, 35, 39], as well as CSFPP with a cut-off ≥ 4 mmHg [15] as predictive parameters of SR. These should be used in conjunction with PPPA [29], PpL [29], PLIV [58], and IE [2], for which future research must identify optimal cut-off values. For TT, TUG is reported as the most accurate, specifically an absolute time (≥ 5.6-s improvement) is advised [73], although there was no clear consensus on which parameter is best. The authors also recommend that TT should be followed up with an MRI assessing cerebral perfusion post-TT, to improve its sensitivity [24, 64]. The patient’s age, symptom severity, and co-morbidities all influence SR outcomes and should be considered in patient selection [3, 35, 39, 44, 61]. The longer symptoms are present before surgery, the worse the prognosis is [38, 39]; hence, early identification and treatment are critical.

In 2001, Hebb and Cusimano [23] stated that more prospective studies, which shunt iNPH patients regardless of their diagnostic test results, were needed to yield precise overall sensitivities and specificities of each test. Twenty years later, we found that this remains the most significant limitation of the literature. Given that in many studies the results of the diagnostic test influence the decision to shunt, accurate false-negative values are difficult to ascertain. We emphasize the importance that future research must aim to shunt all patients to allow for highly valid conclusions on diagnostic efficiency to be derived.

Radiology is also pivotal in the diagnosis of iNPH; however, until recently, studies have not been able to show an association between radiological features and SR [33]. The iNPH Radscale may potentially play an important role in SR prediction and is a promising new tool in the iNPH patient workup [32]. However, due to its relatively recent introduction in 2017, there is currently insufficient literature evaluating its use. Although Kockum et al. [32] have reported 98.5% predictive accuracy of NPH Radscale, another study found it to be unable to predict motor outcomes post-shunting [35]. Nonetheless, the literature on the role of radiology in iNPH is extensive; hence, the authors believe that a separate meta-analysis on the role of radiological tools in the prediction of SR is required. Additionally, biochemical markers have been hypothesized to be valuable in the diagnosis of iNPH. As reported by Leinonen et al. [36], the most important biomarker indicating iNPH may be abnormally low CSF TNF-α concentrations. Nevertheless, this study, as well as a systematic review by Pfanner et al. [50] recently in 2017, reported that no biomarker was able to predict SR. However, Zhang et al. [74] in 2020 reported that studies had found that high concentrations of vascular endothelial growth factor in the CSF may be associated with worse SR. A meta-analysis of both radiology and biochemical markers, together with this meta-analysis, can give more extensive guidance regarding the clinical prediction of SR.

### Limitations

The key limitations of this study were the differences in SR criteria and diagnostic test methodologies between studies. Employing a meta-regression on these independent variables might have increased the validity of our findings. The lack of methodological consistency, as well as not all patients receiving the reference standard (shunt), is reflected in the risk analysis (Fig. 2a) which showed a moderate overall risk of bias for almost half of the studies. The number of studies used in each sub-analysis was moderate (n = 5–7), including more studies may have decreased the risk of sample size bias. Finally, the true diagnostic effectiveness of all diagnostic tests may be higher than reported by the meta-analysis, as many studies either used a medium-pressure valve or did not report using a programmable valve, despite pressure adjustments often necessary postoperatively to avoid over- and underdrainage [7, 28, 36, 46, 47, 69].

## Conclusion

Intraparenchymal ICPM is statistically the most effective diagnostic test, followed by ELD, IT, and lastly TT. The best clinical predictors of SR were found to be a mean ICP wave amplitude $\ge$ 4 mmHg and secondly using a 1- to 4-day ELD with an MMSE cut-off improvement $\ge$ 3. If IT is used, Rout with a cut-off range between 13 and 16 mmHg, in conjunction with a CSFPP cut-off of ≥ 4 mmHg, should be used. Additionally, for TT, a TUG improvement of ≥ 5.6 s can be used. Despite the statistical superiority of ICPM and ELD, the financial cost associated with their use as well as the potential for complications renders it most useful in identifying SR patients with negative IT or TT results. The latter are clinically easier to perform with low complication rates and hence may be used as first-line diagnostic tests to predict SR. When used in addition to diagnostic tests, the severity of symptoms, patient age, and co-morbidities may aid in predicting SR. In the future, standardized methodologies for each diagnostic test and uniform criteria for SR must become the norm to draw better comparisons.

## Abbreviations

*ABP*: Arterial blood pressure
*CIPM*: Continuous intraventricular pressure monitoring
*CO*: Cardiac output
*CSF*: Cerebrospinal fluid
*CSFPP*: CSF pulse pressure
*CT*: Computerized tomography
*ELD*: Extended lumbar drainage
*EVD*: External ventricular drainage
*DOR*: Diagnostic odd ratio
*DSL*: DerSimonian-Laird
*HHS*: Homburg hydrocephalus scale
*ICP*: Intracranial pressure
*ICPM*: Intracranial pressure monitoring
*IE*: Intracranial elastance
*iNPH*: Idiopathic normal pressure hydrocephalus
*iNPHGS*: Idiopathic normal pressure hydrocephalus grading scale
*IT*: Infusion tests
*KI*: Kiefer index
*LIFT*: Lumbar infusion test
*logDOR*: Log diagnostic odd ratio
*MMSE*: Mini mental state examination
*MMT*: Mini mental test
*MRI*: Magnetic resonance imaging
*mRS*: Modified Rankin scale
*NPV*: Negative predictive value
*Pcsf*: Continuous lumbar CSF pressure
*PLIV*: Plateau level of intracranial venous blood volume
*PPPA*: Plateau pulse pressure amplitudes
*PPV*: Positive predictive value
*PpL*: Steady-state plateau pressure
*PRISMA*: Preferred Reporting Items for Systematic Reviews and Meta-Analyses
*PVI*: Pressure volume index
*PWI-MRI*: Perfusion-weighted imaging MRI
*QUADAS*: Quality Assessment of Diagnostic Accuracy Studies
*RAP*: Correlation coefficient between pulse amplitude and ICP
*RAVLT*: Rey auditory verbal learning test
*ROCF*: Rey-Osterrieth complex figure
*Rout*: Resistance to outflow of CSF
*SD*: Standard deviation
*SLHS*: Stein and Langfitt hydrocephalus score
*SPECT*: Single-photon emission computerized tomography
*SR*: Shunt response
*SROC*: Summary receiver operating characteristic
*STT*: Spinal tap test
*TT*: Tap test
*TUG*: Timed up and go test
*VIT*: Ventricular infusion test
*VPS*: Ventriculoperitoneal shunt
*WMS*: Wechsler memory scale
*10St*: 10-M walk in step test
*10Ti*: 10-M walk in time test
*∆Kiefer*: Change in Kiefer score
